# Planning to fail? Credibility and financing of corporate transition plans in hard-to-abate sectors

**DOI:** 10.1016/j.isci.2026.116282

**Published:** 2026-06-08

**Authors:** Sophie Maria Anneke Klein, Friedemann Polzin, Xander Urbach

**Affiliations:** 1 Utrecht University, Copernicus Institute for Sustainable Development, Innovation Studies, Princetonlaan 8a, 3584 CB Utrecht, the Netherlands; 2 Utrecht University, School of Economics (U.S.E.), Kriekenpitplein 21-22, 3584EC Utrecht, the Netherlands; 3 MN Asset Management, Responsible Investment, Prinses Beatrixlaan 15, 2595AK Den Haag, the Netherlands

**Keywords:** Environmental policy, Social sciences, Economics

## Abstract

This study evaluates whether corporate transition plans align with the objectives of the Paris Agreement, highlighting a structural tension between long-term decarbonization goals and the shorter financial planning cycles that influence investment behavior and corporate decision making. Drawing on the firm-level transition plans and financial data of 411 publicly listed companies in hard-to-abate sectors, the analysis assesses carbon performance alignment across near-term 2027/2028, medium-term 2035, and long-term 2050 horizons. The research examines how specific financial characteristics influence the credibility of corporate climate strategies and identifies leverage points for stakeholders to improve transition plan disclosure. The results show that alignment with a 1.5 °C trajectory is particularly weak in the medium term, precisely when transformative emission reductions are most critical. While many firms articulate ambitious climate commitments, these are often insufficiently embedded in financial planning. Stronger alignment between capital expenditure and climate objectives, alongside lower financing costs, is associated with more credible transition strategies.

## Highlights

• A mid-term gap exists in corporate climate alignment with 1.5 °C pathways
• Most corporate climate targets lack a credible financial underpinning
• Alignment of capital expenditure with climate targets results in credible transition plans
• Lower cost of capital relates to better alignment, thus enabling transition activities

## Graphical abstract

**Figure:**
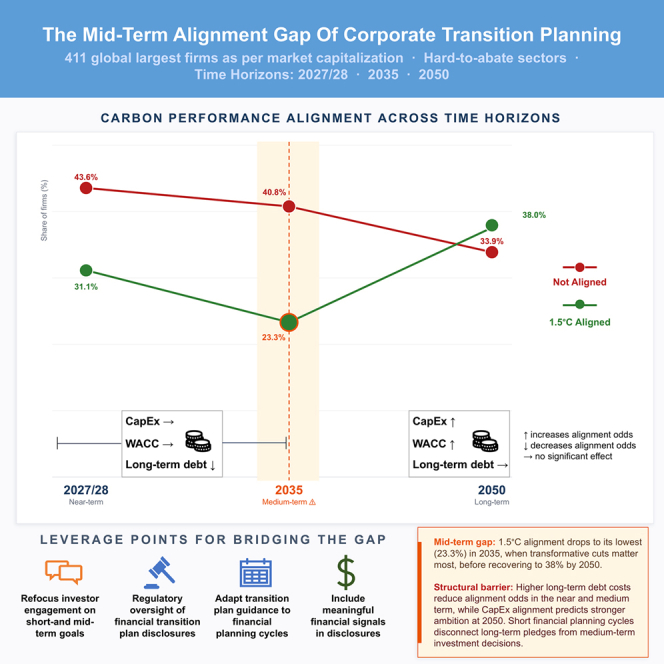

## Introduction

Achieving extensive decarbonization in line with the Paris Agreement requires vast near-term capital investment,^1^^,^^2^ particularly to drive systemic transformations in hard-to-abate sectors that account for a large share of global emissions.^3^^,^^4^ Climate targets can catalyze a fundamental shift in how individual actors approach sustainability, moving beyond incremental gains toward genuine, consistent emission reductions.^5^ While increasing investor and stakeholder pressure has led many firms to adopt long-term climate targets, such an emphasis risks deferring critical near-term action. Recently, scholars have documented that firms fail to meet their climate targets, post-adjust or scale back ambitions.^1^^,^^6^^,^^7^^,^^8^^,^^9^ As the window for effective climate action continues to shrink, near-to medium-term corporate strategies and actions become increasingly urgent, as does the corresponding financial planning required for their implementation.^10^^,^^11^

The transformation of business models, which is especially critical in hard-to-abate sectors,^7^^,^^12^ is a long and complex process that cannot be achieved through incremental emission reduction initiatives alone but requires deep strategic and operational changes, supported by corresponding financial investment.^13^ The complexity lies in planning and financing for the whole transition trajectory, as many firms operate and plan in shorter business cycles.^14^^,^^15^

In response to this tension, new regulations such as the Corporate Sustainability Reporting Directive (CSRD) require firms to develop so-called transition plans.^16^ As a result, a rapidly evolving landscape of corporate transition plans is emerging, making it critical to examine what these plans actually entail.^17^^,^^18^^,^^19^ Transition plans are forward-looking documents intended to align a company’s operations, financial planning, and strategic direction with net-zero emissions pathways.^20^ Unlike traditional sustainability reporting, which often focuses on historical performance and compliance metrics, transition plans function as strategic instruments, outlining a concrete, actionable pathway toward achieving climate targets by 2050.^21^ These plans also aim to identify the capital requirements needed to enable deep structural transformations, such as investments in technological innovation or nature-based solutions.^18^^,^^22^ By linking current sustainability performance with future climate ambitions, transition plans signal strategic priorities and financing needs to stakeholders, particularly investors.^23^^,^^24^

This paper stands at the intersection of the two growing but still only partially integrated literatures of corporate transition planning^25^ and sustainable finance.^26^ While both strands have expanded rapidly, we still know relatively little about how financial conditions influence the credibility over time, investors’ role in shaping those plans, and how the plans can promote more realistic planning. Through the lens of signaling theory,^27^^,^^28^ we conceptualize transition plans as financial signals in a context of pronounced information asymmetry between firms and investors, arguing that the credibility of these plans depends on whether they are reflected in firms’ financial decisions under conditions of uncertainty. This perspective highlights the need to examine not only climate targets but also the financial mechanisms through which such targets are pursued. Hence, this study addresses the following research question: *To what extent do internal capital allocation and external financing conditions shape the credibility of corporate transition plans across different time horizons in hard-to-abate sectors?*

Empirically, we assess the alignment of corporate climate targets with the Paris Agreement’s 1.5 °C objective to limit global average temperature levels over short- (2027/2028), medium- (2035), and long-term (2050) horizons. Drawing on a global dateset of firm-level transition plans and financial data of 411 large publicly listed firms in hard-to-abate sectors, we examine how external financing conditions and internal capital allocations shape credible transition pathways based on the Sectoral Decarbonization Approach (SDA), which provides a scientifically grounded, sector-specific benchmark for assessing whether firm-level targets align with Paris-consistent pathways.^2^^,^^29^ This approach enables us to assess whether financial disclosures support climate targets and to capture sectoral dynamics, given that decarbonization drivers and financing constraints differ markedly across high-emitting sectors.^30^ Offering (financial) stakeholders a more reliable basis for assessing transition credibility than climate targets alone, our analysis focuses on two central mechanisms (internal and external financing): the weighted average cost of capital (WACC) as a market-based assessment of transition credibility and capital expenditure (CapEx) as evidence of internal implementation.

Our results show that many companies set climate targets that are misaligned from the outset with established climate pathways, particularly in the medium term, and that these targets often lack substantive financial underpinning. Although comprehensive financial transition planning in our sample remains limited, we find that CapEx alignment disclosure shows a strong association with long-term carbon performance alignment, while generic green CapEx disclosure shows no significant relationship. The findings indicate that higher financing costs are associated with lower alignment in the medium and long term, pointing toward market recognition of transition risk.

The article makes three important contributions: *Theoreticall*y, we show that transition plans only partially act as credible signals for financial market participants and reduce information asymmetries.^13^^,^^31^ Alignment depends not only on ambition but, critically, also on near- and medium-term capital decisions. Going forward, companies should adapt transition plans to accommodate financial planning and align with real-world corporate planning cycles rather than focusing exclusively on long-term net-zero visions. *Empirically*, we document a medium-term alignment gap, which is the phase in which substantial investments into carbon emission reduction need to take place.^8^^,^^18^ However, few companies disclose whether their CapEx is related to their emission pathways or phasing out of emission-intensive operations. *Methodologically*, this is one of the first papers to build on the forward-looking alignment dataset based on the analyses of the transition pathway initiatives using a sectoral decarbonization approach.^32^ The findings have implications for regulators and standard-setters designing transition plan requirements,^33^^,^^34^ for investors seeking to steer corporate decarbonization through engaging with companies,^26^^,^^35^^,^^36^ and for firms integrating climate targets into core financial strategy.^37^

The remainder of this paper is structured as follows: We first review the relevant literature and then present our data and empirical approach. Subsequently, we report the results on carbon performance alignment and its financial determinants and, finally, discuss the findings and identify leverage points for policy, investors, and firms.

### Literature review

Forward-looking transition plans can be understood as market-facing commitments that address information asymmetry between firms and investors.^24^ Managers hold non-public information about the feasibility, timing, and financing needs of decarbonization, particularly under uncertainty, so climate targets alone are weakly informative about implementation. Signaling theory explains how organizations convey credible information to reduce information asymmetry in financial reports^27^ and is well established in the corporate finance literature, also considering sustainability aspects in this stream.^38^ A multitude of external pressures drives the transparency and accuracy of companies’ carbon performance disclosures.^39^ To gain legitimacy, companies signal credibility to investors and stakeholders through standardized financial reporting and sustainability disclosures.^27^^,^^28^

As investors and other external stakeholders cannot observe the true quality of the ambition to decarbonize, they must rely on clear, observable signals to judge the reliability and veracity (i.e., the integrity or genuineness) of the signaler.^27^ Transition plans represent costly signals, as data collection and projections into the future require human and technical systems and are not easily counterfeited. However, as they have been introduced only recently, the “signal fit” needs to be established, that is, “the extent to which the signal is correlated with unobservable quality.”^27^ Signaling plays a crucial role in attracting investment for companies’ transition activities, with nonfinancial reporting and disclosure being key determinants of strategic investment decisions.^31^^,^^40^

In this context, transition plans are informative not simply because they articulate long-term climate targets but also because they may reveal whether those targets are reflected in financial decisions.^18^ We therefore conceptualize the credibility of transition planning as the degree of alignment between stated climate objectives and observable financial behavior. When transition plans are decoupled from capital allocation and financing conditions, they risk remaining largely rhetorical; when they are embedded in financial decision-making, they function as substantive signals to markets.^19^

Empirically, firms must be financially unconstrained to invest in transitional environmental and social objectives^41^ and disclose the resulting carbon emissions.^42^ The decision to disclose financial information on transition planning, for example, in the Carbon Disclosure Project (CDP) questionnaire, is influenced by the availability of financial resources, such as profitability and leverage (i.e., the ratio between debt and total capital), as well as preexisting sustainability management and reporting systems that reduce preparation costs.^39^^,^^43^ Greater leverage increases pressure on management to disclose carbon information, yet highly leveraged firms may withhold key carbon risk data to protect their negotiating position.^44^ Together, this suggests that both the disclosure and implementation of transition plans are further shaped by firms’ financial conditions rather than reflecting climate ambition alone.

### Hypotheses development

To assess the credibility of transition planning empirically, we focus on two complementary financial mechanisms. First, WACC, which reflects the average cost a firm faces to raise capital through debt and equity and is a decisive factor in investment decisions,^45^^,^^46^ captures external financing conditions shaped by sector risk, leverage, firm characteristics, and market conditions. While WACC primarily reflects these structural factors, climate transition risk and carbon exposure have been shown to influence financing costs in equity and debt markets.^47^ Prior empirical research on transition alignment suggests that the level of alignment is not consistently priced by markets, but that uncertainty around transition credibility is.^48^ Companies require resources at low costs to finance the required capital-intensive investments in low-carbon products and services. If the financial market acts on the (mis)alignment of transition plans, we expect a lower cost of capital for the ambitious firms.^49^

*H1: WACC will be negatively associated with carbon performance alignment*

Second, CapEx captures internal implementation, because capital allocation reveals whether stated climate targets are translated into real investment decisions.^50^^,^^51^ Where financial actions are disclosed, they are linked to financing outcomes. Firms with stronger, financially grounded net-zero transition disclosure components exhibit lower loan spreads, suggesting that markets reward transition commitments backed by concrete financial actions.^19^^,^^34^ CapEx alignment thus serves as a costly signal of credible commitment by linking stated climate targets to observable investment decisions, distinguishing substantive transition plans from rhetorical commitments.^25^ Therefore, we derive our second hypothesis:

*H2: Disclosing CapEx will be positively associated with carbon performance alignment*

Together, WACC and CapEx offer complementary perspectives on transition plan credibility: WACC reflects external validation through financing conditions, whereas CapEx reflects internal enactment through capital allocation. This dual perspective allows us to test whether transition plans meaningfully bridge the temporal gap between long-term climate targets and near- and medium-term financial decisions.

## Results

### The mid-term gap: Patterns of corporate carbon performance alignment

**Figure 1:**
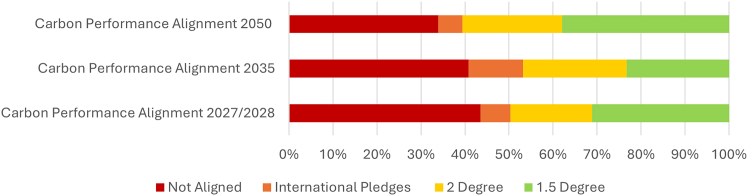
Distribution of carbon performance alignment categories across 2027/2028 (*N* = 411), 2035 (*N* = 412), and 2050 (*N* = 419) CPA is a categorical variable ranging from 0 (not aligned) to 3 (1.5°C aligned). Source: TPI V5.0 (2024).

Corporate climate ambition is uneven across time horizons, with higher near-term commitments, a concerning drop in medium-term ambition, and only partial increased ambition by 2050, revealing structural misalignment and lack of ambition (Figure 1; Tables S3 and S4 for sectoral overview and descriptive statistics).

In 2027/2028, approximately 31% of firms are aligned with a 1.5 °C pathway, whereas 44% remain unaligned. In the medium term (2035), the share of firms aligned with a 1.5 °C pathway declines to 23%, with more firms shifting toward international pledges or 2 °C alignment. This reduction in ambition emerges precisely during the period when deep decarbonization becomes essential. It is at this stage that firms encounter complex challenges, including addressing Scope 3 emissions, reorganizing supply chains, and undertaking broader business model transformation.

By 2050, alignment with a 1.5 °C pathway rises again to 38%, slightly surpassing the 2027/2028 level. However, more than one-third of firms remain unaligned, indicating persistent long-term uncertainty and implementation barriers despite increasing investor pressure to publish long-term climate goals. While many firms demonstrate short-term ambition, a substantial share still lack coherent medium- and long-term transition pathways, consistent with evidence that firms are not currently on track to meet their stated emissions targets.^1^

**Figure 2:**
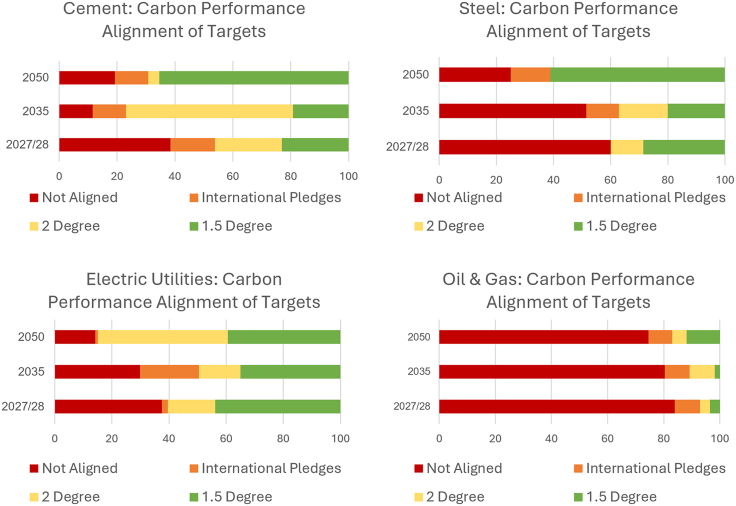
Distribution of carbon performance alignment categories for selected high-emission sectors for 2027/2028, 2035, and 2050 Sectors shown (2050): cement (*N* = 26), steel (*N* = 36), oil & gas (*N* = 59), and electricity utilities (*N* = 99). CPA is a categorical variable ranging from 0 (not aligned) to 3 (1.5°C aligned). Source: TPI V5.0 (2024).

Sectoral alignment trajectories diverge markedly, reflecting differences in structural challenges, technological readiness, and strategic engagement (Figure 2). *Cement* shows the most pronounced improvement over time, with 1.5 °C alignment rising from 23% in 2027/28 to 65% by 2050. *Steel* demonstrates moderate increases in ambition; the sector advances from 28% 1.5 °C alignment in 2027/28 to 61% by 2050. The shift reflects growing momentum toward transition, likely supported by emerging low-carbon technologies. *Electricity utilities* follow a gradual path. While 1.5 °C alignment slightly declines by 2035, the share of unaligned firms drops significantly by 2050. *Oil and gas* remains the least aligned sector across all years. Starting with just 4% 1.5 °C alignment in 2027/28 and reaching only 12% by 2050, the sector shows minimal improvement, pointing to persistent structural and strategic resistance to deep decarbonization.

### Financing of transition plans: The missing cornerstone

A credible transition plan requires integration with financial strategy, particularly in the near and medium term. To assess their financial credibility, we use two key variables: WACC, capturing external financing conditions, and CapEx, reflecting internal investment in transition-related activities.

**Figure 3:**
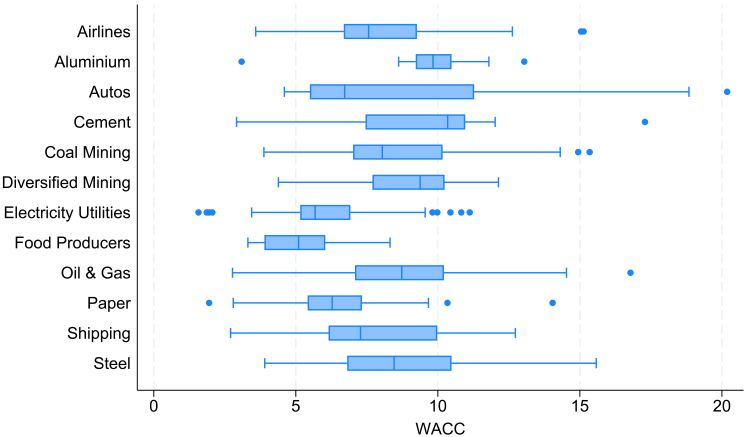
Weighted average cost of capital varies substantially across hard-to-abate sectors WACC by sector in percentage (*N* = 396), as a share of all firms disclosing carbon performance alignment. Boxplots display the median (center line), interquartile range (box), and outside values (dots). Source: Thomson Reuters Eikon (2024) and TPI V5.0 (2024).

WACC reflects broader macro-financial constraints and sectoral risk perceptions. Companies with a higher WACC signal more expensive financing, which may hinder investments in long-term, capital-intensive, low-carbon technologies. A lower WACC suggests more favorable financing conditions and greater capacity to pursue transition investments.^41^ Firms facing higher WACC may delay or deprioritize decarbonization due to cost barriers, whereas those with lower WACC are more likely to engage in strategic, forward-looking planning. WACCs differ significantly across sectors as Figure 3 shows, with electricity utilities, autos, and paper exhibiting the lowest financing costs.

CapEx reflects how firms invest in long-term assets and infrastructure. When aligned with climate goals, CapEx signals a shift toward transition activities, such as renewable energy, electrification, and hydrogen technologies, while phasing out unabated fossil fuel operations.^51^ We examine three dimensions: green CapEx (investment in future green or sustainable projects), CapEx alignment (consistency between capital spending and climate objectives), and CapEx for phase-out (plans to end unabated fossil fuel activities by a target date). Overall, up to 20% of the firms in our sample report having green CapEx. Capital allocation alignment or phase-out is less prevalent, with less than 5% of firms, except in the electricity utility sector. Notably, capital allocation disclosure showed the strongest sectoral concentration: 45% of missing CapEx alignment data came from oil & gas firms. This pattern suggests strategic non-disclosure, where firms with weaker climate commitments in carbon-intensive sectors are less likely to disclose capital allocation plans. This interpretation aligns with research on selective environmental disclosure.^52^

### Financial determinants of transition plans: Empirical links

**Figure 4:**
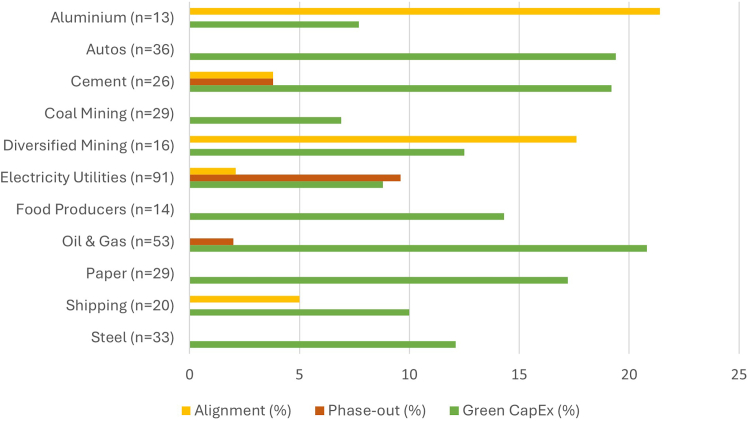
Share of firms disclosing green CapEx, CapEx alignment, and CapEx phase-out by sector (as a share of all firms disclosing carbon performance alignment in percentage) All indicators are binary (0 = not disclosed, 1 = disclosed). In the airlines sector, none of the companies disclosed CapEx-related information. Source: Thomson Reuters Eikon (2024) and TPI V5.0 (2024).

**Figure 5:**
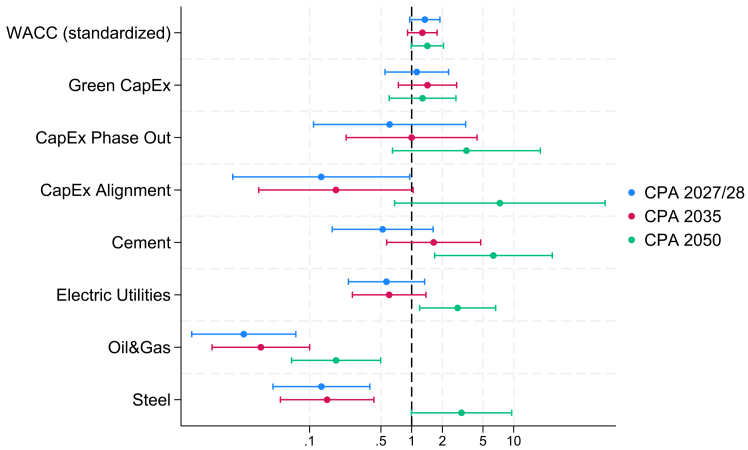
Forest plots of financial and capital expenditure characteristics as determinants of carbon performance alignment Ordered logit odds ratios with 95% confidence intervals for CPA 2027/2028, 2035, and 2050 (*N* = 368–369 per model). Controls include geography, sector, market capitalization, and ESG emissions score. ∗*p* < 0.10, ∗∗*p* < 0.05, and ∗∗∗*p* < 0.01. Error bars represent 95% confidence intervals. See Table S6. Source: Thomson Reuters Eikon (2024) and TPI V5.0 (2024).

Our main analysis links financial characteristics to target alignment, revealing limited use of CapEx for transition activities (Figure 4). However, where CapEx alignment is reported, it appears to be a robust predictor of strong ambitions for carbon performance alignment goals (Figure 5). To quantify this effect, predicted probability calculations show that CapEx alignment disclosure increases the probability of 1.5°C alignment in 2050 by 32.3 percentage points (*p* = 0.021)—the largest substantive effect among all predictors and comparable to firm size (26.9 pp, *p* = 0.052). Notably, this substantially exceeds other capital allocation disclosures: phase-out plans show a 21.1 percentage point increase (*p* = 0.082), while green CapEx disclosure shows only 8.0 percentage points (*p* = 0.200), suggesting that comprehensive forward-looking alignment disclosure matters more than backward-looking green investment reporting.

The findings also suggest that WACC functions as both a barrier and a lever for transition depending on the sectoral context (Table S5 for sectoral effects; Table S6 for regressions). Moving from low to high cost of capital (2-SD change) corresponds to an 11.7 percentage point increase in alignment probability (*p* = 0.029), while improved ESG emissions scores show a 9.9 percentage point increase (*p* = 0.021). The patterns reveal significant sectoral variation, with sectors such as oil and gas, steel, autos, and coal mining among the least aligned, whereas shipping and diversified mining exhibit a higher alignment compared to our baseline (airlines). The analysis also shows significant regional variation, revealing higher alignment among European firms compared to those in other regions.

#### Hypothesis 1: External financing perception (WACC)

Correlation analysis reveals a negative association between WACC and carbon performance alignment in 2050 (*r* = −0.15, *p* <0 .01), suggesting that firms with lower financing costs demonstrate stronger alignment. However, the regression analysis controlling for sector, geography, and firm characteristics reveals a markedly different pattern: WACC shows a positive relationship with alignment that strengthens over time, becoming statistically significant by 2050 (*OR* = 1.48, *p* <0 .05), suggesting that a 1 standard deviation increase in financing costs is associated with 48% higher odds of alignment. This indicates that within sectors and regions, higher financing costs are associated with higher odds of Paris-aligned targets, reflecting sectoral heterogeneity in how financing costs influence transition planning (Table S4). Interestingly, our analysis using the cost of long-term debt as an alternative indicator reveals a 22% decrease in the likelihood of being in a higher alignment category (Table S11); hence, lower financing costs are related to higher alignment. This partly confirms hypothesis 1.

While higher WACC generally constrains capital-intensive decarbonization investment, it operates differently in highly carbon-intensive sectors. In electricity utilities and oil and gas specifically, higher financing costs are associated with stronger alignment, likely because elevated capital costs make long-payback fossil fuel projects economically less attractive relative to lower-risk renewable investments. In practice, WACC is shaped by numerous factors, including sector risk, capital structure, firm size and broader market conditions. The sector-specific relationship we identify suggests that in carbon-intensive industries with substantial locked-in assets, financing costs may influence the relative economics of transition versus business-*as*-usual strategies. For example, oil and gas exploration projects can span over a decade and carry elevated impairment risk. Oil and gas firms investing in renewable power generation achieved an average return on capital employed of 6%, comparable to traditional oil and gas projects.^53^

Reinforcing this shift, Equinor, an international energy company, applied a 3% discount rate for impairment testing of US offshore wind assets in Q2 2025, lower than prevailing US Treasury yields,^54^ showing how perceived risk profiles differ between fossil and renewable investments within the same firm. These findings indicate that the role of financing costs in transition planning cannot be understood in a uniform way. Rather, WACC’s influence depends fundamentally on industry structure, asset composition, and the relative economics of decarbonization pathways available to firms in various sectors.

#### Hypothesis 2: Internal financing strategy (CapEx)

##### CapEx alignment

CapEx alignment emerges as a critical internal financing strategy associated with transition planning, but the relationship proves temporally complex. Correlation analysis reveals no significant associations in the near and medium term, but a positive correlation emerges by 2050. Moreover, regression analysis shows firms disclosing CapEx alignment have significantly lower odds of alignment in 2027/28 and 2035 (a 90% decrease in likelihood of being in a higher category) but higher odds by 2050 (Figure 5; Table S6). To assess relative variable importance, we examined both statistical significance and economic magnitude. CapEx alignment shows the largest effect size (OR = 9.56 for 2050), indicating that firms with aligned CapEx are nearly 10 times more likely to achieve 1.5 °C alignment.

Firms aligning CapEx with long-term low-carbon pathways are substantially more likely to set aligned climate targets for 2050. This temporal reversal warrants careful interpretation. CapEx alignment disclosure remains rare, limiting the robustness of near-term estimates. The negative associations in the near and medium term may reflect implementation challenges faced by early adopters or indicate that firms disclosing CapEx alignment are being transparent about ongoing misalignment. However, the strong positive effect in 2050 suggests that long-term capital commitment serves as a credible signal of strategic intent.

At the same time, case-based evidence underscores the challenges firms face in aligning capital investments with long-term climate strategies. For instance, the Swiss-based cement company Holcim, a recognized sustainability leader, maintains science-based targets initiative (SBTi)-validated targets for both the near and long term and has published a forward-looking CapEx plan referencing the EU Taxonomy in which Holcim commits over 4 billion Swiss francs through 2032 in support of its decarbonization strategy.^55^ The company follows European Sustainability Reporting Standards (ESRS) guidance, disclosing CapEx alignment over a minimum 5-year and maximum 10-year horizon.^56^ Even this frontrunner does not employ long-term capital commitments, illustrating why so few firms disclose long-term CapEx alignment despite its apparent value in signaling credible transition intent.

##### CapEx phase-out

CapEx phase-out shows the weakest effects among the internal financing indicators. Correlation analysis reveals no significant effects in the near and medium term, though a weak positive correlation emerges for 2050. Regression results reveal no statistically significant effects at any time horizon (Table S6). These findings suggest that current efforts to divest from high-emission assets show limited association with carbon performance alignment. For CapEx phase-out to meaningfully support decarbonization, it likely needs to be combined with reinvestment or innovation strategies rather than pursued as a stand-alone approach.

Companies that commit to phasing out unabated fossil fuel-related activities often fail to disclose CapEx plans beyond a single business cycle. This lack of long-term financial planning helps explain the weak correlation between CapEx phase-out and broader transition indicators. For instance, Enel, an Italy-based global utility, reaffirms its commitment to phase out coal-fired power generation by 2027 and thermal generation by 2040.^57^ It also aims to end gas sales to customers by 2040, with intermediate targets to increase electricity sales. While these goals are validated by SBTi and confirmed by TPI as aligned with a 1.5 °C scenario, Enel does not disclose CapEx plans extending beyond 2027.

These findings highlight a tension between strategic ambition and operational flexibility. CapEx phase-out signals long-term intent but often lacks financial detail for assessing credibility. CapEx alignment, while offering concrete evidence of transition efforts, is typically constrained by short planning horizons. Even highly aligned firms rarely commit beyond a standard business cycle, favoring flexibility in capital budgeting. Taken together, we find some evidence in support of hypothesis 2.

##### Green CapEx

Green CapEx, while primarily backward-looking and less indicative of forward transition planning, serves more as a signal of whether any transition-related financing is already in place. Correlation and regression analyses show no significant relationship between green CapEx and carbon performance, also across sectors. These patterns imply that green CapEx may offer value when strategically deployed in high-emission industries.

For example, one frontrunner with long-term 1.5 °C targets and an almost aligned 2035 target, the Australia-based steel company BlueScope Steel, has committed green CapEx and discloses forward-looking plant-by-plant investments,^58^ yet it remains unclear when CapEx in unabated, carbon-intensive assets will cease, underlining the sectoral complexity of the energy transition in the steel industry and the limited availability of near-term, cost-efficient decarbonization levers. CapEx alignment signals tangible climate action, distinguishing substantive investments from symbolic commitments.

## Discussion

### Theoretical implications

Our results indicate that many firms in hard-to-abate sectors remain on trajectories, even in their planning, that will likely fall short of their stated carbon performance ambitions and credible implementation. Even where climate targets appear aligned with long-term climate objectives,^24^^,^^29^ the absence of concrete operational mechanisms, intermediate milestones, and clearly articulated financial provisions substantially weakens their credibility as transition signals, that is, reflecting true intentions to decarbonize.^13^^,^^59^

We show that this reflects a deeper structural mismatch between long-term decarbonization objectives and corporate financial planning. Firms articulate carbon performance targets across multiple time horizons, yet their near- and medium-term capital allocation and financing arrangements frequently diverge from these trajectories. This disconnect underscores the inherent complexity of deep business transformation in incumbent, capital-intensive industries and the persistent tension between short corporate planning cycles and the sustained investment required for a net-zero transition.^14^^,^^50^ The result is further supported by the long-term debt cost specification, which shows that higher debt financing costs are associated with lower near- and medium-term alignment, consistent with evidence that financially constrained firms may invest less in transitional objectives.^41^

This misalignment is consequential from a signaling perspective,^27^ as transition plans are meant to function as credible signals to financial markets. When these plans are decoupled from observable financial behavior, they risk being interpreted as largely rhetorical rather than substantive commitments.^31^^,^^40^ Accordingly, our findings suggest that the current format of many transition plans does not yet provide investors with sufficiently reliable information about how firms will operationalize their climate strategies.

Against this backdrop, our study advances debates on climate target setting and transition finance in three ways. First, we provide a forward-looking empirical assessment of the credibility of corporate transition planning rather than relying solely on historical emissions or stated targets, extending the prior literature.^8^^,^^13^^,^^18^ Second, we demonstrate how both internal capital allocation (CapEx) and external financing conditions (WACC) shape the feasibility and quality of transition pathways in sector-specific and temporally varying ways, revealing that the relationship between financial characteristics and transition credibility is more complex than previously recognized.^25^^,^^34^ Third, we deepen understanding of financial signaling in sustainability transitions and clarify the role that institutional investors, standard setters, and regulators can play—through engagement, capital allocation, and stewardship—in steering firms toward more coherent and actionable transition strategies.^26^

Our missing data diagnostics reveal that capital allocation disclosure itself is informative. Firms withholding CapEx alignment information exhibited significantly lower climate performance, with 45% of non-disclosure concentrated in oil & gas, the sector facing the greatest transition pressure. This pattern suggests disclosure is strategic: firms confident in their transition plans reveal capital commitments, while laggards remain silent. This finding has two implications. First, the absence of CapEx disclosure should be interpreted as a negative signal rather than neutral missing information, consistent with voluntary disclosure theory. Second, as mandatory climate disclosure regimes expand globally (EU CSRD, SEC climate rules), tracking changes in disclosure patterns among current non-disclosers will reveal whether regulatory mandates successfully improve transparency among laggards. Our results establish a baseline of strategic non-disclosure under voluntary frameworks against which future mandatory disclosure can be evaluated.

In terms of generalisability, while our sample focuses on large, publicly listed firms in hard-to-abate sectors, key findings suggest broader applicability. The structural tension between climate targets and financial cycles reflects fundamental mismatches that affect organizations across contexts. Substantial geographic variation reveals higher alignment among European firms subject to CSRD and ESRS requirements, though gaps remain even in these leading jurisdictions.^56^ Current guidance mandates ambitious emissions reduction targets without requiring corresponding financial planning and targets only large firms.^11^ Moreover, under the auspices of competitiveness or protectiveness, regulation can further amplify this mismatch and favor incumbent industries by hollowing out sustainability regulation or supporting fossil incentives^60^

The sector-specific WACC dynamics we identify, among which are higher financing costs, make carbon-intensive projects less viable in oil and gas. These likely stem from fundamental asset impairment economics that are applicable where similar risks exist, consistent with evidence that financing costs reflect market uncertainty around transition credibility rather than alignment per se.^48^ Furthermore, there are technologies depending on fossil fuels that remain hard-to-abate and, as such, have a higher cost tolerance. Managers can prohibit transition capital investment if the incentives do not align with compensation, adding a competing explanation to the “mid-term gap”.^61^ The gap may narrow as business practices and regulatory oversight evolve, although underlying tensions will likely persist between typical business cycles and decarbonization timelines.^1^

### Leverage points for transition planning

#### Standard-setters should adapt transition plan guidance to accommodate financial planning

The tension between long-term carbon alignment and shorter-term financial planning limits transparency and weakens accountability. Because companies in hard-to-abate sectors may require flexible strategies, they feel less inclined to transparently report forward-looking financial plans.^2^ At the same time, plans should reflect whether there is a genuine effort at business transformation.^8^ Our analysis suggests that ambition level may not be the sole reason for misaligned carbon performance and financial planning; the empirical evidence shows that even companies with aligned climate objectives struggle with CapEx disclosures on a timescale longer than five years.

Transparency challenges are compounded by methodological constraints in alignment assessments.^11^^,^^29^ For instance, the TPI evaluates firms based on forward-looking CapEx data to 2050, yet firms often follow regulatory guidance, such as the ESRS, that does not require disclosure beyond a 10-year timescale. The absence of long-term forecasts may reflect strategic caution rather than a lack of ambition, given the inherent uncertainty of projecting decades ahead. Therefore, we argue for aligning transition plan expectations with short-and medium-term financial planning, which is more actionable and better reflects business decision-making cycles.

#### Firms should include meaningful financial signals in transition plan disclosures

Transition plans can provide a meaningful signal, both facilitating financial support for firms’ implementation efforts and investor engagement for steering their portfolio to meet climate targets.^18^^,^^26^^,^^28^ Firms should prioritize integrating climate targets into core capital allocation processes to signal credible transition commitment. Our findings show that CapEx alignment is the only capital measure significantly associated with long-term carbon performance alignment, yet very few firms in our sample disclose this information. This lack of disclosure undermines the signaling function that transition plans are intended to serve, and evidence suggests that debt markets increasingly price the credibility of financial climate commitments.^18^^,^^19^ Companies serious about transitioning should commit to aligning future CapEx with stated climate targets and strategies, even if constrained to near- and medium-term action. Additionally, firms should provide a discussion of how financing conditions shape their sector’s transition strategies. Our analysis reveals that the relationship between cost of capital and carbon performance varies fundamentally across industries.^30^ In carbon-intensive sectors, particularly, firms should transparently discuss how financing costs inform strategic choices between traditional and low-carbon investments. For instance, oil and gas companies could explain how project economics differ between fossil fuel exploration and renewable investments under prevailing capital costs.^62^ This context-specific disclosure moves beyond generic net-zero commitments toward substantive explanation of the financial mechanisms enabling or constraining the transition and consequently enhances the credibility of the transition plan.

#### Investors should refocus financial engagement on short- and medium-term goal setting

Based on our findings, financial institutions should refocus engagement from long-term net-zero targets to short- and especially medium-term commitments.^26^ The 2027–2035 period is a critical window when deep decarbonization becomes urgent, yet corporate alignment is weakest. Investors should move beyond evaluating headline climate targets to assessing financial credibility. CapEx alignment is the only financial measure significantly associated with long-term carbon performance, yet less than 1% of firms disclose this information. Investors should prioritize firms that integrate climate objectives into capital allocation decisions rather than rewarding ambitious targets unsupported by financial planning.^18^ Additionally, engagement strategies must recognize sector-specific dynamics; in carbon-intensive industries, higher financing costs may incentivize rather than constrain decarbonization. Finally, the financial sector should work with standard-setters and governments to overcome fragmented expectations. Companies receive conflicting messages from investors, even within coalitions, undermining effectiveness in steering climate performance.^63^ Financial institutions can “build the field” and work with standard-setters and governments to foster greater alignment between policy (e.g., ESRS) and practice (e.g., TPI).

#### Regulators should establish binding oversight mechanisms for transition plan disclosures

Our findings show geographic variation, with European firms subject to CSRD and ESRS requirements demonstrating significantly higher alignment at near- and medium-term horizons. This suggests that mandatory frameworks can influence corporate behavior, although alignment gaps persist even in leading jurisdictions, indicating that disclosure requirements alone are insufficient without enforcement. The case for mandatory requirements is reinforced by our finding that firms withholding CapEx disclosure showed lower carbon performance alignment. Starting in 2025, companies originally subject to the Non-Financial Reporting Directive must report following ESRS for the financial year 2024, yet scrutiny is left to the member-states, and many have not translated ESRS requirements into binding law, leaving oversight to “best-practice” guidance.^64^ Although transition plan guidance has been available since the adoption of the Paris Climate Agreement,^2^ EFRAG’s *State of Play 2025* concedes, “Notable variation remains across both countries and sectors.”^65^ Regulators should establish concrete enforcement mechanisms by designating competent authorities to review transition plan quality, requiring independent assurance of forward-looking targets and financial data, and imposing penalties for non-compliance. Jurisdictions beyond Europe should incorporate these lessons when implementing transition plan requirements, ensuring disclosure mandates are accompanied by credible oversight from the outset rather than relying on market discipline alone to ensure quality and credibility.

### Suggestions for further research

Based on our findings, future research could examine the internal strategic mechanisms that shape corporate transition planning and explain the observed temporal differences in climate ambition. In particular, more work is needed to understand how firms integrate financial decision-making into their transition strategies, including the role of finance teams in capital allocation, risk assessment, and long-term planning. Comparative research across regulatory contexts would also be valuable to assess how different policy frameworks shape corporate transition planning. Moreover, further research could employ a longitudinal research design to capture the financial indicators’ evolution over time.

### Limitations of the study

This article provides an initial assessment of forward-looking corporate transition plans and their financial foundations, yet several limitations warrant consideration. Although the TPI framework represents an advance over self-reported climate data, alignment assessments rely on long-term net-zero ambitions that reflect intended rather than realized performance, which may lead to an overestimation of firms’ actual transition progress. In addition, forward-looking CapEx data beyond a five-year horizon is rarely disclosed, constraining the precision of long-term projections. Regarding methodology, while the SDA framework accounts for structural differences across sectors, heterogeneity in value chain exposure and decarbonization costs may still hinder full comparability between firms. Moreover, multiple methodologies exist for setting and evaluating science-based targets, and results can vary depending on the chosen approach.^2^ Nonetheless, the consistent application of the SDA in this study supports a reasonable level of comparability across firms.

Inherent to every dataset, methodological assumptions, and subjective classifications can impact the assessment. While errors should be filtered out through companies reviewing their own scores, methodological assumptions can in some cases misclassify results by over- and under-classifying alignment. For example, TPI will not assess carbon performance alignment targets from oil and gas companies that do not specify, on a mid-term and long-term timescale, the specific actions they expect from customers.^66^ Rather, the companies that do not mention customer actions as a prerequisite for their emissions target will be assessed. Obviously, besides changing their product supply, all companies in the oil and gas sector depend on customers to change demand from fossil fuels to bio-based and renewable energy. Therefore, companies with long-term net zero targets align with the TPI methodology, while other companies that simply mention their dependency on customers do not. Finally, although the TPI dataset offers a uniquely detailed and forward-looking perspective on corporate carbon alignment, its coverage remains yet limited relative to the broader population of high-emitting companies, and some firms might strategically decide to be selctive in disclosure.

## Resource availability

### Lead contact

Further information requests should be directed to the lead contact, Friedemann Polzin (f.h.j.polzin@uu.nl).

### Materials availability

This study did not use or generate reagents.

### Data and code availability

• The data are available upon request from the lead contact.
• The code on which the analysis is based is included (Stata 18 do-file).
• Any additional information required to reanalyze the data reported in this paper is available from the lead contact upon request.

## Acknowledgments

We are very grateful to the two anonymous reviewers for their insightful and constructive comments, which significantly improved the quality of this work. We also extend our sincere thanks to the editor for their guidance and support throughout the publication process, and to Cătălina Papari and Jonatan Pinkse for their friendly review. Earlier versions of this work were presented at the GRONEN Research Conference 2024 and EURAM 2025, as well as at an internal section seminar at Utrecht School of Economics (U.S.E.), a Research Exchange Workshop with the Centre for Sustainable Business at King's College London, and an internal workshop at MN Asset Management in 2025. We thank the participants for their valuable feedback and engaging discussions.

## Author contributions

S.K. contributed to all stages of the research. F.P. supported the conceptualization, data analysis, and manuscript review. X.U. was involved in the framing and development of the discussion and the identification of leverage points.

## Declaration of interests

The authors declare no potential conflicts of interest with respect to the research, authorship, and/or publication of this article.

## Declaration of generative AI and AI-assisted technologies in the writing process

During the preparation of this work, the authors used Claude to support brainstorming and language editing. After using these tools or services, the authors carefully reviewed and edited the content and take full responsibility for the content of the publication.

## STAR★Methods

### Key resources table

**Table:** 

REAGENT or RESOURCE	SOURCE	IDENTIFIER
**Deposited Data**		

Corporate transition plan data	Transition Plan Initiative	https://www.transitionpathwayinitiative.org/corporates V.5
Financial data	Eikon Refinitiv	https://eikon.refinitiv.com/
Financial data	FactSet	https://www.factset.com/
Corporate transition plan data (for robustness check)	Carbon Disclosure Project	https://www.cdp.net/en/data
*Software*		
Statistical software	Stata 18	Do-file for the analysis (Data S1)

**Other**		

Sectoral decarbonization approach and methodology	Transition Plan Initiative	https://www.transitionpathwayinitiative.org/corporates TPI’s methodology report

### Experimental model and study participant details

Omitted as our study does not involve biological models.

### Method details

#### Research design

To answer our research question “To what extent do internal capital allocation and external financing conditions shape the credibility of corporate transition plans across different time horizons in hard-to-abate sectors?” this study uses a quantitative hypothetico-deductive research design.^68^ To the best of our knowledge, we are the first to link internal and external financing conditions to a forward-looking measure of sustainability performance and analyze sectoral patterns of alignment.^32^ We cannot claim that financing conditions are causing potential misalignment; neither can we rule out the opposite, hence this study is mainly analyzing associations between the two.^69^ Due to the novelty of the measure and lack of time series data, we perform a cross-sectional analysis, taking the assessment as of the carbon alignment as an indicator for future performance. Based on the conceptual considerations in the sustainability disclosure and corporate finance literature we derived the main models and included control variables commonly used^38^^,^^70^ (Table S1).

#### Data sources

The study used the latest version (V5.0) of the Transition Pathway Initiative (TPI) database. The dataset covers the largest 2,000 firms by market capitalisation in carbon intensive sectors, such as food, oil and gas, mining and transportation. Firms were selected based on data completeness, global representation across five continents and availability of carbon performance assessments. TPI assessments are based exclusively on publicly available information, and analysts benchmark companies across sectors and time horizons, aligning their transition strategies with global climate goals. The TPI dataset is a novel source of forward-looking carbon performance information and, to our knowledge, no other dataset provides more comparable scenario-based alignment measures at the firm level.^32^ Financial data were sourced from Thomson Reuters Eikon and FACTSET, including green CapEx, WACC, market capitalisation and leverage ratios (Table S1 for a variable overview). All continuous financial variables were standardized using z-scores prior to analysis to facilitate comparability across firms and models.^69^ Outliers were visually inspected using boxplots and treated where necessary. WACC and market capitalisation were winsorized at the first and 99th percentiles (Table S7).

##### Dependent variable: carbon performance alignment

Carbon performance is evaluated by examining how firms’ climate targets align with TPI’s sectoral benchmarks, which reflect the diverse decarbonization trajectories and challenges that characterise different sectors.^71^ These benchmarks are based on the SDA, which allocates the global carbon budget to different regions and sectors in line with Paris Agreement targets.^2^ Alternative methodologies exist, including Implied Temperature Rise which translates projected cumulative greenhouse gas emissions into an estimated temperature outcome.^48^ While ITR facilitates portfolio aggregation and risk communication, the SDA approach used here provides sector-specific benchmarks and enables separate assessment across near-, medium-, and long-term horizons, which is essential for examining the temporal dynamics central to our research question. The SDA translates international climate goals into company-specific emissions intensity trajectories. To add nuance to the analysis, we examine the steel, cement, electricity utilities and oil and gas sectors in greater depth, as they are highly emissions intensive and require significant investment for decarbonization. We also provide real-world company examples that illustrate how these dynamics unfold in practice.

Firms’ recent and current emissions intensities are calculated using reported data, whereas future intensities are projected based on disclosed emissions reduction targets. This enables the construction of emissions intensity pathways, which are then evaluated against TPI’s sector specific benchmarks. Three benchmark scenarios are used.

• 1.5 °C scenario reflecting the most ambitious Paris target
• Below 2 °C scenario targeting an approximately 1.65 °C increase
• International or national pledges scenario based on the International Energy Agency’s (IEA’s) 2020 stated policies projection targeting an approximately 2.6 °C increase

Each scenario includes projections for 2027/2028 (near-term), 2035 (medium-term), and 2050 (long-term) horizons. Carbon performance alignment (CPA) is coded as an ordinal variable: 0 = not aligned with any scenario, 1 = aligned with national pledges only, 2 = aligned with below 2°C, 3 = aligned with 1.5°C.

#### Independent variables

Our primary financial variables capture both external financing conditions and internal capital allocation: WACC (Weighted Average Cost of Capital) reflects the average cost a firm faces to raise capital through debt and equity, calculated as the weighted average of the cost of equity and after-tax cost of debt. WACC is a key determinant of investment decisions and captures external financing conditions shaped by sector risk, leverage, firm characteristics, and market conditions.

Capital Expenditure (CapEx) Disclosure captures three dimensions of internal implementation.

• CapEx Alignment: Binary indicator of whether the firm discloses capital expenditure explicitly aligned with climate targets
• Phase-out CapEx: Binary indicator of whether the firm discloses capital expenditure plans to phase out carbon-intensive assets
• Green CapEx: Binary indicator of whether the firm discloses capital expenditure on low-carbon technologies or green investments

Control variables include market capitalization (firm size), ESG emissions score (environmental performance), geographic region, and sector (Table S1 for complete definitions).^38^^,^^70^

#### Missing data treatment

Our analysis employs listwise deletion, resulting in a final sample of 369 firms with complete data for all model variables (88% of the 419 firms with carbon performance assessments). To assess whether this approach introduces bias, we conducted comprehensive missing data diagnostics (Tables S2 and S3). Missing data rates for key variables were modest (5–6%) and revealed systematic patterns inconsistent with random missingness: (1) Firms with incomplete financial data exhibited significantly lower carbon performance alignment than those with complete data (mean CPA = 1.15 vs. 1.71, t = −2.77, *p* = 0.006), (2) Missingness varied significantly across sectors (χ^2^ = 679.45, *p* < 0.001) and (3) Financial variables differed substantially by sector (WACC: F = 7.48, *p* < 0.001; range: 5.2–9.5%).

These patterns indicate Missing Not At Random (MNAR), where missingness is systematically related to the outcome. Under MNAR conditions, imputation methods risk introducing bias by attributing average characteristics to firms that differ systematically from those with complete data.^72^ The observed pattern, lower-performing firms more likely to have missing financial data, suggests that imputation would artificially inflate alignment scores for strategic non-disclosers. We therefore employed listwise deletion, which provides unbiased parameter estimates under MNAR despite reduced precision. This approach is appropriate when analyzing disclosure behavior itself, as imputation would obscure the strategic patterns we seek to understand. With only 5–6% missing data for key variables, the precision cost is minimal relative to validity concerns from inappropriate imputation.

### Quantification and statistical analysis

All analysis was performed with Stata 18; the code and output are provided in the supplementary material (see key resources table). The study employed a combination of descriptive and inferential statistical methods to examine the relationship between financial indicators and firms’ carbon alignment. Due to the forward-looking nature of the data, a cross-sectional research design was applied. Descriptive statistics summarise all variables, and Pearson correlations explore associations and screen for potential multicollinearity (see Tables 1 and 2). Sectoral and regional differences in alignment were explored using ANOVA followed by Tukey post-hoc comparisons^69^ (Tables S4 and S5).

**Table 1:** Summary statistics of main variables

Variable	Count	Mean	Std. Dev.	Min	Max
CPA 2027/2028	411	1.372	1.316	0	3
CPA 2035	412	1.294	1.222	0	3
CPA 2050	419	1.647	1.292	0	3
CDP transition plan	1151	1.56	0.662	0	2
WACC	1011	7.421	2.712	1.576	20.391
Leverage ratio	2009	−0.0150174	0.7395764	−0.4118222	14.93191
WACC long-term debt	1011	0	1	−2.385	9.179
Market capitalization	1919	1,356,816.1	15217694	29.574	5.274e+08
Green CapEx	1023	0.092	0.289	0	1
CapEx phase-out	2026	0.022	0.146	0	1
CapEx alignment	2026	0.005	0.074	0	1
ESG emissions score	1021	71.819	23.597	0	99.917

**Table 2:** Correlation matrix

Variables	(1)	(2)	(3)	(4)	(5)	(6)	(7)	(8)	(9)	(10)	(11)
(1) CPA_2027_28	1.000	–	–	–	–	–	–	–	–	–	–
(2) CPA_2035	0.815∗	1.000	–	–	–	–	–	–	–	–	–
	(0.000)	–	–	–	–	–	–	–	–	–	–
(3) CPA_2050	0.454∗	0.674∗	1.000	–	–	–	–	–	–	–	–
	(0.000)	(0.000)	–	–	–	–	–	–	–	–	–
(4) CDP transition plan	0.142∗	0.140∗	0.355∗	1.000	–	–	–	–	–	–	–
	(0.030)	(0.032)	(0.000)	–	–	–	–	–	–	–	–
(5) WACC	−0.058	−0.084	−0.150∗	−0.081∗	1.000	–	–	–	–	–	–
	(0.248)	(0.094)	(0.003)	(0.042)	–	–	–	–	–	–	–
(6) Market cap	−0.082	−0.085	−0.066	0.031	−0.014	1.000	–	–	–	–	–
	(0.109)	(0.095)	(0.189)	(0.299)	(0.674)	–	–	–	–	–	–
(7) Green CapEx	−0.018	0.047	0.093	0.120∗	−0.048	−0.015	1.000	–	–	–	–
	(0.722)	(0.354)	(0.064)	(0.002)	(0.125)	(0.653)	–	–	–	–	–
(8) CapEx phase-out	−0.017	0.018	0.110∗	0.049	0.016	−0.002	0.102∗	1.000	–	–	–
	(0.743)	(0.727)	(0.028)	(0.100)	(0.612)	(0.925)	(0.001)	–	–	–	–
(9) CapEx alignment	−0.025	−0.030	0.112∗	0.011	0.027	−0.005	0.048	0.035	1.000	–	–
	(0.625)	(0.552)	(0.026)	(0.698)	(0.391)	(0.823)	(0.130)	(0.115)	–	–	–
(10) ESG emission score	0.111∗	0.121∗	0.227∗	0.266∗	−0.019	0.083∗	0.145∗	0.062∗	0.041	1.000	–
	(0.027)	(0.016)	(0.000)	(0.000)	(0.557)	(0.010)	(0.000)	(0.050)	(0.191)	–	–
(11) WACC long-term debt	−0.038	−0.071	−0.152∗	−0.220∗	0.464∗	−0.042	−0.082∗	−0.015	−0.014	−0.082∗	1.000
	(0.447)	(0.160)	(0.002)	(0.000)	(0.000)	(0.202)	(0.009)	(0.630)	(0.653)	(0.009)	–

Ordered logit models^67^ were estimated separately for the 1.5 °C, below 2 °C, and national pledges scenarios for the years 2027/2028, 2035, and 2050 (Tables S6 and S7). These estimators assume proportional odds ratios, that is, the “effort” it takes for a company to move from a non-aligned to a 2 °C pathway is the same as achieving 1.5 °C when the firm is on the 2 °C pathway. Statistical significance thresholds for the analysis are: ∗*p* < 0.10, ∗∗*p* < 0.05, and ∗∗∗*p* < 0.01.

#### Robustness checks

We validated the proportional odds assumption underlying our ordered logit models through multiple approaches. Model fit statistics show consistent patterns across time horizons (Pseudo R^2^ ranging from 0.204 to 0.262). We attempted to relax the proportional odds assumption via Brant test and multinomial logit.^73^ Both approaches did not converge due to perfect separation from rare predictors, demonstrating that the proportional odds constraint is necessary for model stability. The model exhibits satisfactory fit statistics (AIC: 786.67; BIC: 1021.32; Table S9 for all models). Multicollinearity diagnostics further support model validity, with variance inflation factors ranging from 1.11 to 5.92 and a mean VIF of 1.82, indicating no evidence of problematic multicollinearity^69^ (Table S8). The results across all specifications remain consistent with our main findings, supporting the robustness of the identified relationships.

We conducted several robustness checks to validate and extend our findings. First, we substituted our primary dependent variable with an alternative transition plan evaluation measure from the CDP dataset (Table S10), which assesses whether organisations have a climate transition plan aligned with a 1.5 °C world. Second, we tested alternative specifications of our key financial variables: an alternative measure of the WACC using the long-term cost of debt (Table S11) as well as leverage ratio as an alternative financial constraint measure (Table S12). Third, we employed a binary dependent variable that distinguished fully aligned firms from all others, which also relaxes the strong assumptions of the proportional odds ratios (ORs) of the ordered logistic regression, confirming robustness to alternative operationalisations of carbon performance alignment (Table S13).

## Supplemental information

Document S1. Tables S1–S13

Data S1. Stata 18 do-file for the analysis

## References

[1] Jiang X., Kim S., Lu S. (2025). Limited accountability and awareness of corporate emissions target outcomes. Nat. Clim. Change.

[2] Krabbe O., Linthorst G., Blok K., Crijns-Graus W., van Vuuren D.P., Höhne N., Faria P., Aden N., Pineda A.C. (2015). Aligning corporate greenhouse-gas emissions targets with climate goals. Nat. Clim. Change.

[3] Griffin P., Heede C.R. (2017).

[4] Newell P.J., Geels F.W., Sovacool B.K. (2022). Navigating tensions between rapid and just low-carbon transitions. Environ. Res. Lett..

[5] Bjørn A., Fantke P., Jolliet O., Laurent A., Owsianiak M., Ryberg M., Hauschild M., Vea E.B. (2026). Beyond net zero climate targets: a research agenda for absolute environmental sustainability assessment to support decisions at different scales. Environ. Res. Lett..

[6] Avidan M., Walls J.L., Dowell G.W., Cauderay V. (2026). Temporal (In)consistency in Sustainability Disclosure. J. Bus. Ethics.

[7] Dietz S., Gardiner D., Jahn V., Noels J. (2021). How ambitious are oil and gas companies’ climate goals?. Science.

[8] Rogelj J., Geden O., Cowie A., Reisinger A. (2021). Three ways to improve net-zero emissions targets. Nature.

[9] Ruiz Manuel I., Blok K. (2023). Quantitative evaluation of large corporate climate action initiatives shows mixed progress in their first half-decade. Nat. Commun..

[10] Falduto M.R.C., Rocha M. (2020).

[11] Rekker S., Ives M.C., Wade B., Webb L., Greig C. (2022). Measuring corporate Paris Compliance using a strict science-based approach. Nat. Commun..

[12] Rezaeian M., Pinkse J., Rigby J. (2024). Transforming titans: The role of policy mixes in business model adaptation strategies for sustainability transitions. Energy Res. Social Sci..

[13] Hale T., Smith S.M., Black R., Cullen K., Fay B., Lang J., Mahmood S. (2022). Assessing the rapidly-emerging landscape of net zero targets. Clim. Policy.

[14] Pinkse J., Demirel P., Marino A. (2024). Unlocking innovation for net zero: constraints, enablers, and firm-level transition strategies. Ind. Innovat..

[15] Slawinski N., Bansal P. (2015). Short on Time: Intertemporal Tensions in Business Sustainability. Organ. Sci..

[16] Ballesteros F., Hessenius M., Hüttel A., Marchewitz C., Neuhoff K., Schütze F., Stolle L. (2023). Climate transition plans: State of play in EU legislation and policy recommendations. Research Platform Sustainable Finance, Policy Brief 3/2023.

[17] Bolton P., Kacperczyk M. (2026). Firm Commitments. Manag. Sci..

[18] Dikau S., Robins N., Smoleńska A., van’t Klooster J., Volz U. (2025). Prudential net zero transition plans: the potential of a new regulatory instrument. J. Bank. Regul..

[19] Zhou X., Williams R., Shrimali G. (2024). Corporate Net Zero Transition and Financing Cost: Evidence of Impact from Global Energy and Utilities Sectors.

[20] European Commission (2023). Unveiling Biodiversity-Impact Sectors - European Commission. https://green-business.ec.europa.eu/news/unveiling-biodiversity-impact-sectors-2023-04-27_en.

[21] Schütze F., Ballesteros F., Hüttel A., Kempa K., Neuhoff K., Shrimali G. (2024). Enhancing Comparability and Credibility of Transition Plans and Transition Risk Assessment with Standardized Net Zero Scenarios. Research Platform Sustainable Finance, Policy Brief 2/2024.

[22] Griffiths S., Sovacool B., Iskandarova M., Walnum H.J. (2025). Bridging the gap between defossilization and decarbonization to achieve net-zero industry. Environ. Res. Lett..

[23] Babcock A., He A., Ramani V. (2022). Building Investor Trust in Net Zero. J. Applied Corp. Finance.

[24] Bolay A.-F., Bjørn A., Patouillard L., Weber O., Margni M. (2024). What drives companies’ progress on their emission reduction targets?. J. Clean. Prod..

[25] Rose A., Shrimali G., Halttunen K. (2025). A framework for assessing and managing dependencies in corporate transition plans. iScience.

[26] Crona B., Peterson G., Meacham M., Parlato G., Lade S.J., Rocha J.C., Galaz V. (2025). A systems approach to sustainable finance: Actors, influence mechanisms, and potentially virtuous cycles of sustainability. iScience.

[27] Connelly B.L., Certo S.T., Ireland R.D., Reutzel C.R. (2011). Signaling Theory: A Review and Assessment. J. Manag..

[28] Busch T., Scheitza L., Bauckloh T., Klein C. (2025). Does Shareholder Signaling Matter for Corporate Strategy? A Study of ESG- and Carbon-Related Proposals. Organ. Environ..

[29] Bjørn A., Lloyd S., Matthews D. (2021). From the Paris Agreement to corporate climate commitments: evaluation of seven methods for setting ‘science-based’emission targets. Environ. Res. Lett..

[30] Kartal M.T., Magazzino C., Taşkın D., Depren Ö., Ayhan F. (2025). Efficiency of green bond, clean energy, oil price, and geopolitical risk on sectoral decarbonization: Evidence from the globe by daily data and marginal effect analysis. Appl. Energy.

[31] Andrus J.L., Callery P.J., Grandy J.B. (2023). The Uneven Returns of Transparency in Voluntary Nonfinancial Disclosures. Organ. Environ..

[32] Kayral İ.E., Bozkurt M.A., Gontijo T.S., de Souza Groppo G., Goliatt L., Günar A., Saygın D., Başarır Ç. (2026). AI and Climate-Smart Policies for Blue Economy: Eco-Digital Futures.

[33] Chenet H., Ryan-Collins J., van Lerven F. (2021). Finance, climate-change and radical uncertainty: Towards a precautionary approach to financial policy. Ecol. Econ..

[34] D’Arcangelo F.M., Kruse T., Pisu M., Tomasi M. (2023). Corporate Cost of Debt in the Low-Carbon Transition: The Effect of Climate Policies on Firm Financing and Investment through the Banking Channel.

[35] Kölbel J.F., Heeb F., Paetzold F., Busch T. (2020). Can Sustainable Investing Save the World? Reviewing the Mechanisms of Investor Impact. Organ. Environ..

[36] Velte P. (2023). Which institutional investors drive corporate sustainability? A systematic literature review. Bus. Strat. Environ..

[37] Siew D., Hillis L. (2022). Corporate Climate Transition Plans: A Guide to Investor Expectations.

[38] Gillan S.L., Koch A., Starks L.T. (2021). Firms and social responsibility: A review of ESG and CSR research in corporate finance. J. Corp. Finance.

[39] He R., Luo L., Shamsuddin A., Tang Q. (2022). Corporate carbon accounting: a literature review of carbon accounting research from the Kyoto Protocol to the Paris Agreement. Account. Finance.

[40] Hahn R., Reimsbach D., Wickert C. (2023). Nonfinancial Reporting and Real Sustainable Change: Relationship Status—It’s Complicated. Organ. Environ..

[41] Hartzmark S.M., Shue K. (2022). Counterproductive Sustainable Investing: The Impact Elasticity of Brown and Green Firms. Preprint.

[42] Siddique M.A., Akhtaruzzaman M., Rashid A., Hammami H. (2021). Carbon disclosure, carbon performance and financial performance: International evidence. Int. Rev. Financ. Anal..

[43] Ott C., Schiemann F., Günther T. (2017). Disentangling the determinants of the response and the publication decisions: The case of the Carbon Disclosure Project. J. Account. Publ. Pol..

[44] Luo L., Tang Q. (2014). Does voluntary carbon disclosure reflect underlying carbon performance?. J. Contemp. Account. Econ..

[45] Frank M.Z., Shen T. (2016). Investment and the weighted average cost of capital. J. Financ. Econ..

[46] Polzin F., Sanders M., Steffen B., Egli F., Schmidt T.S., Karkatsoulis P., Fragkos P., Paroussos L. (2021). The effect of differentiating costs of capital by country and technology on the European energy transition. Clim. Change.

[47] Bolton P., Kacperczyk M., Samama F. (2022). Net-Zero Carbon Portfolio Alignment. Financ. Anal. J..

[48] Latino C., Shrimali G. (2026). Forward-Looking, Still Divided: Implied Temperature Rise and Cost of Capital. Social Science Research Network.

[49] Velte P., Stawinoga M., Lueg R. (2020). Carbon performance and disclosure: A systematic review of governance-related determinants and financial consequences. J. Clean. Prod..

[50] Berg F., Heeb F., Kölbel J.F. (2022). The Economic Impact of ESG Ratings.

[51] Egli F., Polzin F., Sanders M., Schmidt T., Serebriakova A., Steffen B. (2022). Financing the energy transition: four insights and avenues for future research. Environ. Res. Lett..

[52] Marquis C., Toffel M.W., Zhou Y. (2016). Scrutiny, Norms, and Selective Disclosure: A Global Study of Greenwashing. Organ. Sci..

[53] IEA (2023). https://www.iea.org/reports/the-oil-and-gas-industry-in-net-zero-transitions/executive-summary.

[54] Equinor (2025). Our quarterly results. Financial statements and review.

[55] Holcim (2024). Annual Report 2024: Overview. https://www.holcim.com/investors/publications/annual-report-2024.

[56] ESRS (2024). ANNEX I to Commission Delegated Regulation (EU) 2023/2772 supplementing Directive 2013/34/EU of the European Parliament and of the Council as regards sustainability reporting standards. https://xbrl.efrag.org/e-esrs/esrs-set1-2023.html.

[57] Enel (2024). INTEGRATED ANNUAL REPORT 2024.

[58] BlueScope (2024). Climate Action Report 2024, p. 38.

[59] Saleh H., Battiston S., Monasterolo I., Barreau T., Tankov P. (2026). Estimating firms’ emissions from asset level data helps revealing (mis)alignment to net zero targets. Nat. Commun..

[60] Stegemann H. (2026). Off the drip: Why Europe’s energy crisis response is backwards. https://www.triodos.com/en/articles/2026/off-the-drip-why-europes-energy-crisis-response-is-backwards.

[61] Ritz R.A. (2022). Linking Executive Compensation to Climate Performance. Calif. Manag. Rev..

[62] IAE (2023). https://www.iea.org/reports/the-oil-and-gas-industry-in-net-zero-transitions.

[63] Hastreiter N. (2024). Can investor coalitions drive corporate climate action?. https://www.lse.ac.uk/geography-and-environment/research/discussion-paper-series.

[64] AFM (2025). Sustainability reporting and the Corporate Sustainability Reporting Directive (CSRD). https://www.afm.nl/en/sector/themas/duurzaamheid/csrd.

[65] EFRAG (2025). State of Play 2025 Implementation of the European Sustainability Reporting Standards (ESRS): Observed Practices Based on Statements Issued as of 20 April 2025. https://www.efrag.org/en/news-and-calendar/news/efrag-launches-esrs-statistics-and-report-portal-on-the-2025issued-esrs-sustainability-statements.

[66] Sharp J., Gardiner D., Dietz S., Bouckaert H. (2024). Assessment Framework: Net Zero Standard for Oil and Gas - Transition Pathway Initiative. https://www.transitionpathwayinitiative.org/publications/2024-net-zero-standard-for-oil-and-gas-assessment-framework.

[67] Fullerton A.S. (2009). A Conceptual Framework for Ordered Logistic Regression Models. Socio. Methods Res..

[68] Mellinger C., Hanson T. (2016).

[69] Hair J. (2010).

[70] Coelho R., Jayantilal S., Ferreira J.J. (2023). The impact of social responsibility on corporate financial performance: A systematic literature review. Corp. Soc. Responsib. Environ. Manag..

[71] Dietz S., Bienkowska B., Jahn V., Hastreiter N., Komar V., Scheer A., Sullivan R. (2023). TPI’s Methodology Report: Management Quality and Carbon Performance.

[72] Little R.J., Rubin D.B. (2019).

[73] Brant R. (1990). Assessing Proportionality in the Proportional Odds Model for Ordinal Logistic Regression. Biometrics.

