# Supplementary material for: Planning to fail? Credibility and financing of corporate transition plans in hard-to-abate sectors
Source: iScience. 2026 Jun 8;29(6):116282. doi: 10.1016/j.isci.2026.116282 (PMC13264030; doi:10.1016/j.isci.2026.116282)
Supplement: Document S1. Tables S1–S13 [file mmc1.pdf]

iScience, Volume *29*

Supplemental information

Planning to fail? Credibility and financing of corporate transition plans in hard-to-abate sectors

Sophie Maria Anneke Klein, Friedemann Polzin, and Xander Urbach

Supplemental information

Variables description

Table S1. Variable Overview for the main regression models and robustness checks.

Category	Variable Name	Description	Source	Type
**Dependent variables**	**Carbon Performance 2027-208**	Carbon performance alignment for 2027/2028 based on sectoral benchmarks [1,2]. (Categories: not aligned, international pledges, 2-degree aligned or 1.5-degree Paris-aligned).	TPI	Categorical
	**Carbon Performance 2035**	Carbon performance alignment for 2035 based on sectoral benchmark [1,2]. Idem	TPI	Categorical
	**Carbon Performance 2050**	Carbon performance alignment for 2050 based on sectoral benchmark [1,2]. Idem	TPI	Categorical
**Independent variables**	**CapEx Alignment**	Indicates whether the company commits to align future capital expenditures with long-term GHG targets or the 1.5 °C goal, and discloses the alignment methodology.	TPI	Binary
	**CapEx Phase-Out**	Indicates whether the company has a time-bound commitment to phase out capital expenditures in carbon-intensive assets or products.	TPI	Binary
	**Green CapEx**	Capital expenditures on future green or sustainable projects. [3]	Thomson Reuters Eikon	Binary
	**WACC**	Weighted Average Cost of Capital. [4,5].	Thomson Reuters Eikon	Numeric
**Robustness check**	**CDP Climate transition planning (Alternative Dependent Variable)**	Organization's strategy include a climate transition plan that aligns with a 1.5 °C world.<br>No influence and no plan (0), Influenced but no plan within two years (0), Influenced and developing a plan within two years (1), Yes (2)	Carbon Disclosure Project (CDP)	Categorical
	**Leverage Ratio ((Alternative Independent Variable)**	Ratio of a company's debt to its equity. [6]	Thomson Reuters Eikon	Numeric

	**WACC Long-term debt (Alternative Independent Variable)**	Long-term debt used to finance significant investments (e.g. infrastructure)	Thomson Reuters Eikon	Numeric
**Controls** [7,8]	**Sector Code**	Numeric code representing industry sectors.	TPI	Categorical
	**Geographical Location**	The country of the location of a company's headquarters, which comes from FTSE Russell.	TPI	Categorical
	**Market Capitalization**	The total value of a company's outstanding shares, calculated as share price times number of shares.	Factset	Numeric
	**ESG emission score**	Company current ESG emission score in %	Thomson Reuters Eikon	Numeric

Analysis of Missing Data

Table S2. Missing data rates for key variables.

Variable	**N Missing**	**% Missing**
WACC	23	5.5%
Market Capitalization	26	6.2%
ESG Emissions Score	17	4.1%
Green CapEx Disclosure	17	4.1%
CapEx Alignment Disclosure	22	5.3%
CapEx Phase-out Disclosure	22	5.3%
Sectoral Distribution of Missing Data		
Sector	**WACC Missing (%)**	**CapEx Alignment Missing (%)**
Oil & Gas	30.4%	45.5%
Electricity Utilities	26.1%	18.2%
Steel	13.0%	13.6%
Diversified Mining	8.7%	4.5%
Other sectors	21.8%	18.2%

Table S3. Tests for missing not at random (MNAR).

**Test**	**Statistic**	**p-value**	**Interpretation**
CPA difference by data completeness (t-test)	t = -2.77	0.006	Incomplete-data firms have lower CPA (1.15 vs 1.71)
Sectoral variation in missingness (χ^2)	χ^2 = 679.45	<0.001	Missingness differs significantly across sectors
WACC variation across sectors (ANOVA)	F = 7.48	<0.001	WACC ranges from 5.2% to 9.5% by sector

Note: Sample includes 419 firms with carbon performance assessments. MNAR tests indicate missing data is systematically related to climate performance and varies by sector, supporting listwise deletion over imputation methods.

Analysis of sectoral differences

Table S4. ANOVA analysis for sectoral differences CPA and financials, related to Figure 3

Variable	F-statistic	p-value	Bartlett p	Equal variance
CPA 2027_28	9.07	<0.001	<0.001	No
CPA 2035	9.79	<0.001	0.001	No
CPA 2050	12.38	<0.001	0.006	No
WACC	7.48	<0.001	<0.001	No
GreenCapEx	1.17	0.306	0.052	Yes
LeverageRatio	11.33	<0.001	<0.001	No
CapEx Alignment	3.87	<0.001	<0.001	No
CapEx Phase-out	1.92	0.030	<0.001	No

Note: Most noteworthy pairwise sector differences for WACC (only those with p < 0.05)

Table S5. Sectoral comparison of WACC differences (Tukey post-hoc, significant pairs only, p < 0.05), related to Figure 3 and Figure 4.

Sector Comparison	Difference	95 percent CI	Significant?
Electricity Utilities vs Airlines	−2.07	[−3.84, −0.30]	Yes
Electricity Utilities vs Aluminium	−3.57	[−6.23, −0.90]	Yes
Electricity Utilities vs Autos	−2.61	[−4.39, −0.82]	Yes
Electricity Utilities vs Cement	−2.98	[−4.98, −0.98]	Yes
Electricity Utilities vs Consumer Goods	−2.72	[−5.16, −0.28]	Yes
Electricity Utilities vs Paper	−2.61	[−5.00, −0.21]	Yes
Oil & Gas vs Electricity Utilities	+2.73	[1.17, 4.29]	Yes
Oil & Gas vs Food Producers	+3.65	[0.94, 6.36]	Yes
Steel vs Electricity Utilities	+2.61	[0.78, 4.44]*	Yes
Paper vs Cement	−2.46	[−4.89, −0.02]	Yes
Food Producers vs Cement	−3.91	[−6.89, −0.92]	Yes
Food Producers vs Steel	−2.71	[−5.85, 0.43]	Borderline

Regression Analysis

Table S6. ordered logistic regression: carbon performance alignment with dependent variables CPA 2027/2028, 2035, and 2050, related to Figure 5.

VARIABLES	(1) CPA 2027/28 OR	(2) CPA 2035 OR	(3) CPA 2050 OR[1]
**Main covariates**			
WACC (standardized)	1.304	1.270	1.484**
	(0.232)	(0.223)	(0.285)
Green CapEx	1.016	1.422	1.672
	(0.381)	(0.488)	(0.669)
Climate phase-out CapEx	0.620	1.093	4.049
	(0.546)	(0.835)	(3.520)
Climate CapEx alignment	0.144*	0.187*	9.562*
	(0.148)	(0.167)	(11.68)
Market cap (standardized)	0.463	1.076	2.463*
	(0.320)	(0.713)	(1.218)
ESG emissions score	1.011*	1.006	1.013**
	(0.00601)	(0.00582)	(0.00584)
**Geography (baseline = Australia)**			
Austria	34.83**	17.88**	7.04e+07
Brazil	6.569**	4.316*	0.856
Canada	3.736	3.205	0.570
Chile	5.993*	4.759	1.004
China	1.587	1.243	0.501
Colombia	8.21e-07	1.613	0.349
Denmark	2.11e+07	6.45e+07	1.43e+07
Finland	15.58**	10.38**	1.749
France	9.751*	4.559	4.020
Germany	4.935*	7.241**	11.89*
Greece	1.67e-07	1.357	2.86e+07
Hong Kong	0.312	0.607	0.508
India	1.128	0.624	0.594
Indonesia	0.902	0.116	0.0110*
Ireland	1.54e+07	3.66e+07	1.047
Italy	3.578	4.425	5.223
Japan	2.887	2.364	4.134*
Malaysia	1.756	1.625	0.588
Mexico	1.714	1.379	2.645
Netherlands	0.733	3.562	2.35e+07
New Zealand	5.15e+07	1.08e+08	1.31e+08
Nigeria	9.71e-08	3.22e-08	6.83e-10

[1] Predicted probability effects on 1.5°C alignment (2050): CapEx alignment disclosure increases alignment probability by 32.3 percentage points (pp) (p=0.021), market capitalization by 26.9 pp (p=0.052), phase-out CapEx by 21.1 pp (p=0.082), WACC by 11.7 pp (p=0.029), ESG emissions score by 9.9 pp (p=0.021), and green CapEx by 8.0 pp (p=0.200).

VARIABLES	(1) CPA 2027/28 OR	(2) CPA 2035 OR	(3) CPA 2050 OR[1]
Norway	10.46*	15.09**	2.920
Philippines	2.04e-07	5.48e-07	3.38e-08
Poland	1.21e-07	1.492	0.468
Portugal	10.67	9.158	1.056
Saudi Arabia	5.13e-07	0.657	0.604
Singapore	1.79e+07	4.53e+07	1.389
South Africa	2.636	1.369	0.631
South Korea	7.072*	4.464	1.447
Spain	21.73***	15.74**	1.527
Sweden	10.08**	10.68*	0.582
Switzerland	1.177	2.958	7.193
Taiwan	0.284	0.722	1.010
Thailand	1.217	0.843	0.723
Turkey	0.300	1.034	7.72e+06
United Kingdom	5.189*	5.677*	1.236
United States	2.457	2.547	1.874
**Sectors (baseline = Airlines)**			
Aluminium	0.242*	0.303	2.717
Autos	0.311**	0.342**	2.280
Cement	0.460	1.545	6.699***
Chemicals	1.518	1.06e-07	1.50e-08
Coal Mining	0.266**	0.0458***	0.0601***
Diversified Mining	2.796	3.975*	1.699
Electricity Utilities	0.498	0.529	2.423**
Food Producers	0.319	0.355	1.816
Oil and Gas	0.0195***	0.0230***	0.124***
Paper	0.158***	0.390*	1.402
Shipping	5.613**	1.625	14.85***
Steel	0.0858***	0.101***	4.222**
**Observations**	368	369	369

*p <.10, **p <.05, ***p <.01. Note: WACC and market capitalization are z-standardized.

Table S7. Ordered logistic regression, dependent variables CPA 2027/28, 2035, 2050 (outlier treated); Independent variables WACC and Market capitalization winsorized at 1st and 99th percentiles, related to Figure 5.

VARIABLES	(1) CPA 2027/28 OR	(2) CPA 2035 OR	(3) CPA 2050 OR
**Main covariates**			
WACC (standardized, winsorized)	1.330	1.260	1.504**
	(0.251)	(0.232)	(0.305)
Green CapEx	0.999	1.413	1.703
	(0.376)	(0.485)	(0.679)
Climate phase-out CapEx	0.627	1.094	3.993
	(0.553)	(0.836)	-3.472

Climate CapEx alignment	0.145*	0.186*	9.560*
	(0.149)	(0.166)	(11.69)
Market cap (standardized, winsorized)	0.257	1.065	24.66*
	(0.258)	-1.119	(41.75)
ESG emissions score	1.011*	1.006	1.013**
	(0.00601)	(0.00583)	(0.00583)
**Geography (baseline = Australia)**			
Austria	34.50**	17.90**	7.24e+07
Brazil	6.510**	4.324*	0.853
Canada	3.764	3.199	0.575
Chile	6.693*	4.752	0.696
China	1.583	1.250	0.534
Colombia	9.59e−07	1.617	0.127
Denmark	2.01e+07	2.35e+07	1.49e+07
Finland	15.67**	10.35**	1.776
France	9.855*	4.517	4.180
Germany	4.940*	7.169**	12.41*
Greece	1.89e−07	1.356	2.82e+07
Hong Kong	0.307	0.609	0.514
India	1.137	0.627	0.529
Indonesia	1.111	0.119	0.00355***
Ireland	1.04e+07	1.36e+07	1.053
Italy	3.522	4.419	5.370
Japan	3.133	2.308	3.543
Malaysia	1.771	1.620	0.595
Mexico	1.702	1.385	2.625
Netherlands	0.702	3.549	2.47e+07
New Zealand	3.92e+07	3.95e+07	1.33e+08
Nigeria	1.28e−07	1.03e−07	7.04e−10
Norway	10.68*	14.98**	2.760
Philippines	2.28e−07	1.49e−06	3.45e−08
Poland	1.34e−07	1.517	0.473
Portugal	10.75	9.116	1.105
Qatar	3.396	0.294	2.01e−09
Saudi Arabia	5.01e−07	0.655	0.600
Singapore	1.24e+07	1.67e+07	1.443
South Africa	2.587	1.371	0.651
South Korea	8.966*	4.473	0.470
Spain	21.62***	15.68**	1.558
Sweden	10.14**	10.67*	0.578
Switzerland	1.207	2.943	6.567
Taiwan	0.287	0.719	0.989
Thailand	1.220	0.845	0.720
Turkey	0.285	1.051	7.45e+06
United Kingdom	5.175*	5.736*	1.255
United States	2.477	2.536	1.900
**Sectors (baseline = Airlines)**			
Aluminium	0.237*	0.306	2.846
Autos	0.320**	0.350**	2.116
Cement	0.458	1.550	7.004***
Chemicals	1.531	—	—
Coal Mining	0.266**	0.0461***	0.0535***
Diversified Mining	2.753	3.983*	1.733
Electricity Utilities	0.498	0.527	2.492**
Food Producers	0.313	0.356	2.061

Oil and Gas	0.0194***	0.0232***	0.121***
Paper	0.157***	0.389*	1.467
Shipping	5.583**	1.635	14.68***
Steel	0.0847***	0.102***	4.448**
Observations	368	369	369

**p* <.10, ***p* <.05, ****p* <.01.

Robustness checks

Table S8. Multicollinearity diagnostics (variance inflation factors); OLS specification of main model (see Table S6).

Variable Group	Range of VIF Values	Mean VIF	Note
Financial variables:<br>WACC, WACC (long-term debt), Market Capitalisation, Leverage, (z-standardised)<br>CapEx measures, ESG emissions score (not standardised)	1.30 – 2.96	1.82	No multicollinearity concerns
Sector dummies	1.11 – 3.28	1.82	Expected moderate multicollinearity due to categorical encoding; not problematic
Geographic region dummies	1.12 – 5.92	1.82	Higher VIFs for some regions reflect small category sizes; still acceptable
Overall model	Max = 5.92	Mean = 1.82	Well below conventional thresholds; no evidence of problematic multicollinearity

Table S9. Model fit statistics for ordered logistic and logistic regression models (Tables S6, S10, S11, S12, S13).

Model	N	LR chi²	Pseudo R²	AIC	BIC
**Main Model (CPA dependent variable)**					
CPA 2027/28	368	187,67	0,207	840,01	1074,50
CPA 2035	369	198,18	0,204	893,24	1127,89
CPA 2050	369	236,03	0,262	786,67	1021,32
**Robustness Model (CDP dependent variable)**					
CDP Transition Plan (robustness)	596	216,58	0,232	859,73	1171,44
**Alternative WACC Model- Long-term debt cost**					
CPA 2027	368	-359.201	0.209	838.403	1.072.888
CPA 2035	369	-385.606	0.206	891.212	1.125.859
CPA 2050	369	-334.345	0.259	788.689	1.023.337
CDP Transition Plan	596	-357.885	0.234	857.770	1.169.477
**Alternative WACC Model - Leverage Ratio**					
CPA 2027	364	-355.262	0.207	830.525	1.064.354
CPA 2035	365	-382.115	0.204	884.231	1.118.225
CPA 2050	365	-329.963	0.261	779.926	1.013.920
CDP Transition Plan	584	-344.190	0.245	830.381	1.140.644

**Binary Model**					
CPA 2027	305	-144.179	0.270	370.357	522.890
CPA 2035	295	-136.726	0.207	351.453	495.245
CPA 2050	300	-146.657	0.289	373.314	521.465
CDP Transition Plan	528	-256.550	0.227	609.099	814.016

Table S10. Alternative dependent variable (CDP transition plan); ordered logistic regression.

	(1)
**VARIABLES**	**CDP Transition Plan**
**WACC**	1.088
	(0.214)
**Green CapEx**	1.190
	(0.489)
**CapEx Phase-out**	1.422
	(1.122)
**Climate CapEx Alignment**	889,100
	(1.199e+09)
**Market Capitalization**	14.78
	(41.92)
**ESG emissions score**	1.031***
	(0.00638)
**Controls: geolocation, sector numeric**	
**Observations**	596

**p* <.10, ***p* <.05, ****p* <.01. Note: Climate CapEx, almost perfect prediction due to small number of observation

Table S11. Alternative independent variable (long-term debt cost replacing WACC); ordered logistic regression.

	(1)	(2)	(3)
**VARIABLES**	**CPA 2027/28**	**CPA 2035**	**CPA 2050**
**Long-term debt cost**	0.780*	0.774**	0.816
	(0.100)	(0.101)	(0.109)
**Green CapEx**	0.967	1.347	1.520
	(0.362)	(0.461)	(0.606)
**CapEx Phase-out**	0.587	1.020	3.522
	(0.507)	(0.772)	(3.002)
**Climate CapEx Alignment**	0.129**	0.162**	7.721*
	(0.131)	(0.143)	(9.381)
**Market Capitalization**	0.440	1.071	2.356*
	(0.305)	(0.713)	(1.186)
**ESG emissions score**	1.010*	1.005	1.013**
	(0.00605)	(0.00585)	(0.00586)
**Controls: geolocation, sector numeric**			
**Observations**	368	369	369

**p* <.10, ***p* <.05, ****p* <.01

Table S12. Alternative independent variable replacing WACC (Leverage Ratio); ordered logistic regression

	(1)	(2)	(3)
**VARIABLES**	**CPA_2027_28 OR**	**CPA_2035 OR**	**CPA_2050 OR**
**Leverage Ratio**	1.212	1.093	0.911
	(0.271)	(0.233)	(0.198)
**Green CapEx**	0.925	1.336	1.463
	(0.348)	(0.459)	(0.584)
**CapEx Phase-out**	0.753	1.212	4.928*
	(0.660)	(0.917)	(4.426)
**Climate CapEx Alignment**	0.124**	0.153**	7.532*
	(0.125)	(0.136)	(9.147)
**Market Capitalization**	0.431	1.060	2.353*
	(0.299)	(0.701)	(1.181)
**ESG emissions score**	1.012*	1.006	1.013**
	(0.00618)	(0.00597)	(0.00598)
**Controls: geolocation, sector numeric**			
**Observations**	364	365	365

*p <.10, **p <.05, ***p <.01.

Table S13. Alternative dependent variable (1,5 °C alignment vs. aggregated other alignment categories); logistic regression

	(1)	(2)	(3)	(4)
**VARIABLES**	**CPA 2027 BI**	**CPA 2035 BI**	**CPA 2050 BI**	**CDP Transition Plan BI**
**WACC**	1.653**	1.468	1.393	1.121
	(0.376)	(0.359)	(0.325)	(0.229)
**Green CapEx**	0.938	1.112	2.393*	1.230
	(0.505)	(0.613)	(1.258)	(0.525)
**CapEx Phase-out**	0.314	0.834	3.584	1.380
	(0.406)	(0.966)	(4.183)	(1.129)
**Climate CapEx Alignment**	0.264	0.162	13.00*	
	(0.328)	(0.220)	(19.23)	
**Market Capitalization**	0.0752	0.432	11.87	17.12
	(0.183)	(0.870)	(18.44)	(49.81)
**ESG emissions score**	1.003	0.999	1.012	1.029***
	(0.00724)	(0.00751)	(0.00777)	(0.00674)
**Controls: geolocation, sector numeric**				
**Observations**	305	295	300	528

*p <.10, **p <.05, ***p <.01. Note: CDP transition plan BI is omitted due to perfect prediction.

Supplemental references

1. Canal Vieira, L., Longo, M., and Mura, M. (2024). Responding to a Wicked Problem: How Time, Sense of Place, and Organisational Boundaries Shape Companies' Decarbonisation Strategies. Organization & Environment *37*, 6–31. https://doi.org/10.1177/10860266241229226.

2. Dietz, S., Bienkowska, B., Jahn, V., Hastreiter, N., Komar, V., Scheer, A., and Sullivan, R. (2023). TPI's methodology report: Management Quality and Carbon Performance.

3. Roncalli, T. (2024). Portfolio Alignment and Net Zero Investing. Preprint at Social Science Research Network, https://doi.org/10.2139/ssrn.4891271 https://doi.org/10.2139/ssrn.4891271.

4. Bams, D., and van der Kroft, B. (2022). Divestment, information asymmetries, and inflated ESG ratings. Preprint, https://doi.org/10.2139/ssrn.4126986 https://doi.org/10.2139/ssrn.4126986.

5. Chava, S. (2014). Environmental Externalities and Cost of Capital. Management Science *60*, 2223–2247. https://doi.org/10.1287/mnsc.2013.1863.

6. Albuquerque, R., Koskinen, Y., and Zhang, C. (2019). Corporate Social Responsibility and Firm Risk: Theory and Empirical Evidence. Management Science *65*, 4451–4469. https://doi.org/10.1287/mnsc.2018.3043.

7. Coelho, R., Jayantilal, S., and Ferreira, J.J. (2023). The impact of social responsibility on corporate financial performance: A systematic literature review. Corporate Social Responsibility and Environmental Management *30*, 1535–1560. https://doi.org/10.1002/csr.2446.

8. Gillan, S.L., Koch, A., and Starks, L.T. (2021). Firms and social responsibility: A review of ESG and CSR research in corporate finance. Journal of Corporate Finance *66*, 101889. https://doi.org/10.1016/j.jcorpfin.2021.101889.
